# Aberrant Ganglioside Functions to Underpin Dysregulated Myelination, Insulin Signalling, and Cytokine Expression: Is There a Link and a Room for Therapy?

**DOI:** 10.3390/biom12101434

**Published:** 2022-10-07

**Authors:** Evgeniy Svirin, Johannes de Munter, Aleksei Umriukhin, Elisaveta Sheveleva, Allan V. Kalueff, Andrei Svistunov, Sergey Morozov, Susanne Walitza, Tatyana Strekalova

**Affiliations:** 1 Neuroplast BV, 6222 NK Maastricht, The Netherlands; 2 Laboratory of Psychiatric Neurobiology, Institute of Molecular Medicine and Department of Normal Physiology, Sechenov First Moscow State Medical University, 119048 Moscow, Russia; 3 Institute of General Pathology and Pathophysiology, 125315 Moscow, Russia; 4 Research Laboratory for Advanced Studies in Petrochemistry, Chemical Technology and Biotechnology, Ural Federal University, 620002 Yekaterinburg, Russia; 5 Neuroscience Program, Sirius University of Science and Technology, 354340 Sochi, Russia; 6 School of Biological and Medical Physics, Moscow Institute of Physics and Technology, 141701 Moscow, Russia; 7 Department for Child and Adolescent Psychiatry and Psychotherapy, University of Zurich, University Hospital of Psychiatry, 8008 Zurich, Switzerland; 8 Department of Psychiatry and Neuropsychology, School for Mental Health and Neuroscience (MHeNS), Maastricht University, 6200 MD Maastricht, The Netherlands

**Keywords:** major brain gangliosides, neurodevelopmental disorders, neuroinflammation, myelination, insulin receptor signalling, mice

## Abstract

Gangliosides are molecules widely present in the plasma membranes of mammalian cells, participating in a variety of processes, including protein organization, transmembrane signalling and cell adhesion. Gangliosides are abundant in the grey matter of the brain, where they are critically involved in postnatal neural development and function. The common precursor of the majority of brain gangliosides, GM3, is formed by the sialylation of lactosylceramide, and four derivatives of its a- and b-series, GM1, GD1a, GD1b and GT1b, constitute 95% of all the brain gangliosides. Impairments in ganglioside metabolism due to genetic abnormalities of GM-synthases are associated with severe neurological disorders. Apart from that, the latest genome-wide association and translational studies suggest a role of genes involved in brain ganglioside synthesis in less pervasive psychiatric disorders. Remarkably, the most recent animal studies showed that abnormal ganglioside functions result in dysregulated neuroinflammation, aberrant myelination and altered insulin receptor signalling. At the same time, these molecular features are well established as accompanying developmental psychiatric disorders such as attention-deficit hyperactivity disorder (ADHD) and autism spectrum disorders (ASD). This led us to hypothesize a role of deficient ganglioside function in developmental neuropsychiatric disorders and warrants further gene association clinical studies addressing this question. Here, we critically review the literature to discuss this hypothesis and focus on the recent studies on ST3GAL5-deficient mice. In addition, we elaborate on the therapeutic potential of various anti-inflammatory remedies for treatment of developmental neuropsychiatric conditions related to aberrant ganglioside functions.

## 1. Introduction

Gangliosides or sialo-glycolipids are molecules consisting of glycosphingolipid and one or more sialic acid residues. They are ubiquitous in cell membranes in all vertebrates and are involved in many key cellular processes [1]. Gangliosides of the CNS play critical roles in development and function [2,3,4], facilitating neuronal membrane protein organization, signalling and cell adhesion [4,5]. Brain gangliosides also regulate microglia and cytokine-mediated immune responses, including microglial activation, myelination and platelet activation [2,6,7].

The majority of brain gangliosides consist of derivatives of ganglioside GM3 [8]. The ganglioside GM3, which is the precursor of the principal brain gangliosides including GM1, GD1a, GD1b, GD3, GT1b and GQ1b, is generated by alpha-2,3-sialyltransferase 5 (ST3GAL5) or GM3-synthase [8]. Outside of the brain, GM3 is also known to play a role in membrane microdomain functionality of insulin receptors and in the induction of insulin resistance [9]. Serum GM3 levels were found to be significantly elevated in type 2 diabetes patients with severe obesity [10].

Here, we critically review the recent clinical and pre-clinical evidence of the involvement of aberrations in ganglioside metabolism in a range of neuropsychiatric and CNS pathologies, including attention-deficit hyperactivity disorder (ADHD) and autism spectrum disorders (ASD), and review the possible underlying mechanisms, such as neuroinflammation and oxidative stress, insulin signalling dysregulation and myelination abnormalities, as possible pathophysiological patterns resulting from central ganglioside deficiency.

## 2. Impairment of Ganglioside Metabolism in CNS Disorders

Genetic impairments of ganglioside metabolism are associated with several human disorders, most of which affect ganglioside catabolism, and only few families with disruptions of ganglioside biosynthesis have been reported [4]. Aberrant ganglioside catabolism causes lysosomal ganglioside storage diseases, comprising GM1-gangliosidoses and three forms of GM2-gangliosidoses, which are a group of inherited metabolic diseases caused by a deficiency of the different proteins that break down gangliosides [11]. Under normal conditions, gangliosides are catabolized by a group of lysosomal hydrolases, β-hexosaminidases A and B, encoded by genes *HEXA* and *HEXB*, respectively [11]. Besides hexosaminidases, ganglioside GM2 catabolism also depends on GM2 activator protein (GM2-AP), encoded by the *GM2A* gene, which also provides GM2 for hexosaminidases [12].

Depending on the affected gene, *HEXA, HEXB* or *GM2A*, three variants of GM2 gangliosidoses have been identified: Tay-Sachs’s disease, Sandhoff disease and AB Variant GM2 gangliosidoses [13]. The former two are child-onset disorders, usually rapidly progressing and leading to child death before the age of four [11,14,15]. Tay-Sachs’s disease, or GM2 gangliosidosis variants B and B1, is characterized by acute onset at infantile age with manifestations such as seizures, axial hypotonia and regressions in developmental milestones [16,17,18]. In the case of Sandhoff disease, or variant 0, subacute juvenile onset of ataxia, myoclonus, motor regression, psychotic episodes, intellectual disability and progressive clumsiness are usually observed [18,19]. The AB Variant is an adult-onset chronic disease, characterized by dysphagia, muscle atrophy, cerebellar ataxia, dysarthric speech, muscle weakness, manic depression and psychotic episodes [11,20,21]. GM1 gangliosidosis, in turn, is caused by a deficiency of acid β-galactosidase, usually with infantile onset and clinical hallmarks such as dysmorphic face, severe dysostosis, hepatomegaly, inability to sit and muscle hypotonia, and later on, spasticity, visual failure, foam cells in bone marrow, oligo-sacchariduria and muco-polysacchariduria [12]. The substrates of acid β-galactosidase include not only GM1, but also specific oligosaccharides, so that this disease is marked not only by ganglioside storage but also by oligosaccharidosis and mucopolysaccharidosis, with extra-neuronal clinical involvement due to them [12].

Disruptions of ganglioside biosynthesis are found much less frequently and lead to catastrophic neurological deficits, including severe cognitive impairments, seizures and motor and sensory dysfunctions [22,23]. These ganglioside biosynthesis disorders arise from mutations in genes encoding GM3-synthase (*ST3GAL5*) [2,24,25,26] or GM2/GD2 synthase (*B4GALNT1*) [27,28]. Loss-of-function mutations in either of the proteins lead to the complete absence of major brain gangliosides [23,29]. Loss of *B4GALNT1* activity leads to hereditary spastic paraplegia, for which the main clinical hallmark is a child-onset slowly progressing central demyelination with axon loss and lower extremity spasticity with muscle weakness accompanied by cognitive impairments [27,28,30]. Abnormalities in the *ST3GAL5* gene disrupt GM3 synthesis, leading to intellectual disability, microcephaly, seizures, blindness and deafness, somatic growth failure and metabolic syndrome [23,31]. Notably, it was recently shown that, in families with mutations in *B4GALNT1* that do not lead to the complete loss of the protein activity, the neurological phenotype was much milder than in those with complete loss of function [32]. These observations suggest that, besides complete loss of function of ganglioside-synthesizing proteins, there may be intermediate phenotypes with milder symptoms, indicating that alterations in ganglioside metabolism may be overlooked as contributors to other diseases besides gangliosidoses and severe neurologic conditions.

Indeed, emerging evidence suggests that ganglioside deficiency may have other effects in addition to the severe neuropathology observed in individuals with ST3GAL5 and/or B4GALNT1 deficiency. Recent genome-wide association studies (GWAS) have reported an association between SNPs in gene encoding alpha-2,3-sialyltransferase-III (*ST3GAL3*) and factors regulating ganglioside function and the incidence of schizophrenia, attention-deficit/hyperactivity disorder (ADHD) or autism spectrum disorders (ASD). In particular, in a large-scale integrative analysis of GWAS, comprised of 20,183 ADHD cases and 35,191 controls, using a DEPICT analysis of gene prioritization, pathway and tissue/cell type enrichment analysis, *ST3GAL3* was the top gene associated with ADHD (P = 1.19 × 10^−2^) [33]. Furthermore, the homozygous loss-of-function mutation of *SLC39A8*, a well-established schizophrenia biomarker, was shown to result in serum manganese (Mn) abnormalities, a causal factor of glycosyltransferases’ dysfunction. This mechanism was suggested to underlie the pathophysiology of schizophrenia [34]. A more recent GWAS also revealed a relationship between the increased expression of a *ST3GAL3* transcript in the human foetal brain and a risk for ADHD and schizophrenia [35]. Moreover, children with ASD often displayed increased anti-ganglioside antibody levels [36,37], and changes in ganglioside expression have also been proposed as a biomarker for schizophrenia [38].

However, changes in the ganglioside profile in the brain were also associated with Alzheimer’s disease and amyloidosis [39]. In the hippocampi of an APPswe/PS1dE9 transgenic mouse model of Alzheimer’s disease, the progressive downregulation of major gangliosides and upregulation of acetylated and N-acetyl-galactosaminylated gangliosides were observed with the development of the disease from the early to late stages. The authors speculated that such changes are attributed to the inhibition of GD3-synthase activity [39]. GM1 and other gangliosides were shown to stabilize amyloid fibrils by changing their secondary structure [40,41,42].

Recently, a deficiency of gangliosides GM1 and GD1a in peripheral blood mononuclear cells of patients with Parkinson’s disease was found [43]. A deficiency of gangliosides GM1 and GD1a was also found in the brains of patients with Parkinson’s disease; in particular, this deficit was shown in the substantia nigra [44]. Moreover, reduced synthesis of GM1 in fibroblasts was also revealed in patients with Huntington’s disease [45], and distinct ganglioside content changes were found in the n. caudatus, putamen and cerebellum of post-mortem Huntington’s brains [46]. Elevated levels of gangliosides GM1 and GM3 were found in the spinal cords of amyotrophic lateral sclerosis (ALS) patients, along with increased activities of enzymes mediating their hydrolysis [47]. Anti-ganglioside antibodies were found in a patient with ALS attributed to the P525L mutation in the fused in sarcoma (FUS) gene [48]. Thus, alterations of ganglioside metabolism accompany the much broader spectrum of neurological and psychiatric disorders that was accepted until recently. This warrants further clinical and pre-clinical studies on this question; the use of animal models can be of particular value in this context.

## 3. Modelling of Ganglioside Deficiency in Animals

Several animal models of ganglioside deficiency have been generated to date. One of them is *B4galnt1* knockout (−/−) mice [32]. They synthesize GM3 and GD3 but lack all of the GalNAc-bound gangliosides [1]. *B4galnt1*^−/−^ mice display deficits in hindlimb reflex, balance, coordination and muscle strength [49]. They also show axonal degeneration, which resembles the pattern of human spastic paraplegia, caused by *B4GALNT1* mutations [23]. Mice with heterozygous knockout (+/−) of *B4galnt1*, which leads to only partial ganglioside deficiency, display a Parkinson’s disease-like phenotype, which includes motor impairments, dysfunctions in short-term memory and cardiac and gastrointestinal symptoms [50].

Another animal model of ganglioside biosynthesis disruptions is *St3gal5*^−/−^ mice [51]. Unlike *B4galnt1*^−/−^ mice, these mutants only partly re-capitulate clinical abnormalities, which may be attributable to the compensatory synthesis of 0-series gangliosides GD1α and GM1b [52]. However, *St3gal5*^−/−^ mice do lack the major CNS gangliosides GM3, GM1, GD1a, GD3, GT1b and GQ1b and display motor hyperactivity, impulsivity and inattentiveness [53], enhanced insulin sensitivity [51] and platelet activation and neuronal damage following brain trauma [6,54], so this model provides the opportunity to explore the consequences of the brain’s ganglioside deficiency rather than its complete absence [53]. The severest pathology is observed in double knockout *St3gal5*^−/−^/*B4galnt1*^−/−^ mice. They are devoid of any ganglioside derivatives of LacCer and soon after birth develop severe neurodegeneration with impaired axon-glia interactions, hind limb weakness, ataxia and tremors, and die before two months of age. Such deficiency is also associated with increased inflammatory reactions [55].

**Figure 1:**
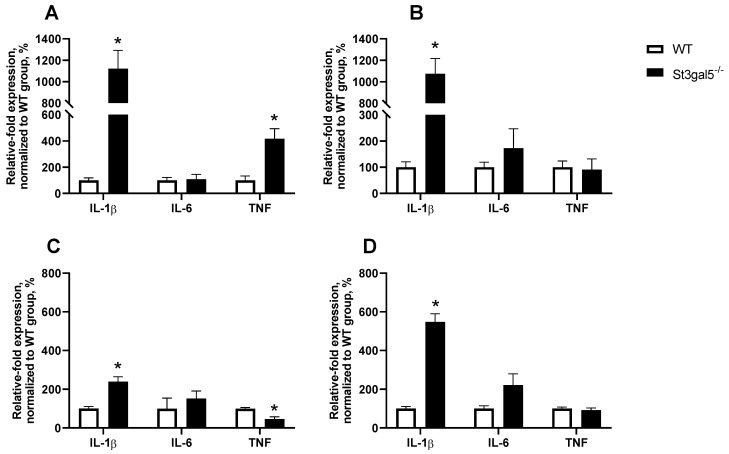
Expression of pro-inflammatory cytokines *Il-1β*, *Il-6* and *TNF* mRNA in (**A**) brains of male and (**B**) female *St3gal5*^−/−^ mice and (**C**) spleens of male and (**D**) female *St3gal5*^−/−^ mice. The expression of *Il-1β* is significantly elevated in both the brain and spleen of both genotypes. Differential mRNA expression of TNF is observed in male and female mutants’ brains, with a significant increase in male mutants compared to controls and the opposite effect in females. Male and female *St3gal5*^−/−^ mice and their wild type littermates, *n* = 6–10 per group, aged 2–3 months, were housed under standard conditions. Mice were euthanized, perfused with saline, and their brains and spleens were dissected and frozen at −70 °C for qPCR assay. Full description of the study can be found in [57]; for primer sequences, see [56]. WT—wild-type mice. * *p* < 0.05 vs. control (wild-type) mice. Data are presented as Mean ± SEM.

Recently, we studied the baseline behaviour of *St3gal5*^−/−^ mice, as data in the available literature was scarce [56]. We have found substantial increases in dominant and neutral social behaviours in male mutants and decreased neutral sociability of female *St3gal5*^−/−^ mice, as well as other behavioural alterations resembling ASD-like and ASD-accompanying features, i.e., repetitive grooming behaviour and increased anxiety. Naïve *St3gal5*^−/−^ male mice exhibited a substantial increase in dominant behaviour, and a similar trend was also found in *St3gal5*^−/−^ female mice. Behavioural alterations were accompanied by increased mRNA expression of pro-inflammatory cytokine interleukin-1β (Il-1β) and tumour necrosis factor (Tnf), both in the prefrontal cortex (PFC) and the spleen of *St3gal5*^−/−^ animals (Figure 1, for methods see [57]).

Additionally, we investigated whether LPS administration causes a different inflammatory response in *St3gal5*^−/−^ mice compared to wild-type controls. We demonstrated profound sex differences in the behavioural reaction to LPS administration. While systemic inflammation led to a substantial increase in dominant and aggressive behaviours in *St3gal5*^−/−^ males, in female mice, an increase in those behaviours was observed in wild-type control mice [56]. Sex differences were also found in the cytokine expression response to LPS administration. In general, compared to wild-type controls, *St3gal5*^−/−^ mice of both sexes had a lower degree of increase in the expression of the pro-inflammatory cytokines in the brain and a higher degree in the periphery. However, the LPS-induced increase of *Il-1β* expression both in the brain and in the periphery was significantly higher in *St3gal5*^−/−^ females than in wild-type controls, while in male wild-type control mice the rise of *Il-1β* expression was higher than in *St3gal5*^−/−^ males [56]. Thus, there are remarkable sex-specific effects of both the *St3gal5* knockout alone and its interaction with inflammatory stress on social and aggressive behaviour. Notably, sex-specific differences in the severity of outcomes were shown for many genetic rodent models of neurodevelopmental disorders [58,59].

As there is a substantial body of evidence suggesting links between brain gangliosides and myelination, we also assessed the mRNA expression of myelination-related proteins, myelin basic protein (MBP), proteolipid protein 1 (PLP1), myelin-associated glycoprotein (MAG) and myelin oligodendrocyte glycoprotein (MOG) in the PFC of naïve *St3gal5*^−/−^ mice. In mutants of both sexes, we showed a nearly two-fold decrease of expression of PLP1 both in mRNA and protein levels. No changes in the expression of the other three myelin proteins were found [56]. Interestingly, a mutation in the *Plp1* gene leading to its decreased expression was found in rabbits with paralytic tremor disease and myelin lipid content in these animals was altered [60]. It was shown that *Plp1*^−/−^ mice, as well as patients with a lack of PLP1, develop length-dependent axonal degeneration progressing with age, with no histological signs of demyelination [61]. Local deficits of axonal transport were also found in the internodes myelinated by PLP1-deficient oligodendrocytes [62]. Given the abovementioned results, we may suggest that decreased expression of PLP1 in the PFC of *St3gal5*^−/−^ mice may arise from aberrant regulation of oligodendrocytes in the lack of major brain gangliosides. A decrease in PLP1 may be a factor contributing to white matter abnormalities affecting brain circuits involved in the regulation of social and aggressive behaviours.

In another study we found increased locomotor activity in mutants of both sexes, as well as elevated anxiety-like behaviour and decreased exploratory behaviour [57]. These changes were accompanied by alterations in the expression of insulin receptor (IR) isoforms in the spleen and liver; in male mice, mRNA expression of both the A and B isoforms of IR was increased in wild type animals compared to mutants in both the liver and spleen, while in females, IR-B expression was increased in the livers of mutants. The body weight of male mutants was significantly increased compared to male control mice. Additionally, we found an increased number of high-amplitude EEG spikes [57], which we speculated to be representative of seizures observed in some of the patients with ganglioside metabolism disorders.

Notably, *St3gal3*^−/−^ mice, which do not lack major brain gangliosides, were shown to have a significantly decreased ratio of myelinated to non-myelinated axons in corpus callosum, as well as reduced MBP protein content [63]. *St3gal3*^−/−^ mice also demonstrated poorer performance in the rotarod test, motor hyperactivity in the open field associated with increased exploratory behaviour and decreased learning in passive avoidance task [63]. Recently, some of the pathological features were also observed in *St3gal3*^+/−^ mice. These mice were shown to have lowered MBP protein expression in the PFC, as well as cognitive deficits in male *St3gal3*^+/−^ mice, and increased activity and enhanced cognitive control in female *St3gal3*^+/−^ mice [64].

It is believed that, while one of the major functions of ST3GAL3 protein is sialylation of glycoproteins, it also participates in synthesis of a minor ganglioside of lacto-series, sialyl-lactotetraosylceramide (sialyl-Lc4) [65], which is found in the brain in concentrations much lower than major brain gangliosides [66]. Additionally, ST3GAL3 also accepts ganglioside GM1 as a substrate [67]. Thus, findings in the *St3gal3* knockout mice together with the suggestion that ST3GAL3 may contribute to the brain minor ganglioside synthesis, further supports the view on possible role of subtle ganglioside metabolism alterations in neuropsychiatric pathology. 

## 4. Gangliosides in Neuroinflammation

Neuroinflammation is known to play a significant role in the pathogenesis of many neuropsychiatric and neurodegenerative disorders. It was implicated as a pathological mechanism in ASD where increases in reactive microglia and astrocytes are found in patients’ brains as well as increased pro-inflammatory cytokines such as IL-1β, IL-6, TNF in brain tissue, cerebrospinal fluid and blood serum [68,69,70,71,72]. For ADHD, developmental exposure to inflammation is considered a risk factor [73] and, in a number of studies, elevated levels of microglia activation markers, pro-inflammatory cytokines and autoantibodies against nervous system cell types were found in ADHD patients, as reviewed in [74]. Developmental neuroinflammation is also viewed as a risk factor for schizophrenia [75,76]. Neurodegenerative disorders such as Alzheimer’s and Parkinson’s diseases [77,78] and ALS [79] are also accompanied by microglial activation and a pro-inflammatory cytokine profile. In turn, gangliosides are known to participate in the regulation of microglia and cytokine-mediated immune responses and platelet activation [2,6,7]. This may suggest a link between ganglioside deficiency and the pathology to which it may contribute.

There is strong evidence of participation of gangliosides in regulation of immune responses in which they have an anti-inflammatory effect [3,80]. The knockout of *B4galnt1* in mice led to elevated infiltrating microglia, and double knockout of *B4galnt1* and GD3-synthase (*St8sia1*) led to the upregulation of inflammation-related genes in the brain, including *Il-1β* and *TNF* [55]. The function of the latter cytokine was also shown to be regulated by gangliosides [81]. Neuro-statin (*O*-acetyl GD1b) [82] and several other *O*-acetylated gangliosides were shown to reduce inducible nitric oxide synthase (*iNOS*) and *Il-6* expression in LPS-activated microglial culture and promote neuronal survival in co-cultures [83], and for microglia treated with lipopolysaccharide (LPS) or IL-1β, the application of gangliosides exerted anti-inflammatory effects [3]. In rats, GM1 administration relieved negative consequences of developmental lead exposure, reverting cognitive deficit and cell death in the hippocampus via an antioxidant effect, as well as activation of the SIRT1/cAMP response element-binding protein (CREB)/brain-derived neurotrophic factor (BDNF) and anti-apoptotic pathways [84,85]. In addition, gangliosides affect nitric oxide synthase (NOS2) and ICAM-1 and MCP-1-mediated signalling [7,86]. On the other hand, major brain gangliosides were shown to play a role in platelet activation in the case of traumatic brain injury or neurodegeneration, leading to enhanced inflammation upon disruption of the blood-brain barrier [54,87]. Thus, while major evidence suggests a strong anti-inflammatory effect of CNS gangliosides, their action seems to be rather complex.

## 5. Gangliosides and Myelination in CNS Pathologies

Evidence links both ganglioside deficiency and neuroinflammation to deficits in myelination. Such deficits are observed in numerous neurological and neuropsychiatric disorders. In addition to well-known demyelinating diseases such as multiple sclerosis and leukodystrophies, neuro-visualization studies found myelination deficits in late-onset neurodegenerative diseases such as Alzheimer’s disease [78,88,89], Parkinson’s disease [78,90] and ALS [91], neurodevelopmental disorders such as ASD and ADHD [92,93,94], and schizophrenia [95]. In addition, factors such as prenatal selective serotonin reuptake inhibitors exposure or social isolation, which are detrimental for CNS development and function, lead to myelination abnormalities, as well as inflammatory disease, e.g., necrotizing enterocolitis in children [96]. Gangliosides are known to participate in myelination in several ways.

One of the major myelination proteins, myelin-associated glycoprotein (MAG), is located on the surface of myelinating glial cells and interacts with neuronal membrane gangliosides to inhibit neurite outgrowth [97]. Gangliosides GD1a and GT1b, which are ligands of MAG, are absent in B4GALNT1 deficiency, and *B4galnt1* knockout mice display axonal degeneration in the CNS and PNS, as well as motor deficits similar to those in *Mag* knockout mice [98]. Finally, the interaction of ganglioside GM1 and MBP stimulates Schwann cell proliferation in the PNS [99]. Recently, we have found decreased mRNA expression of myelination marker *Plp1* in *St3gal5*^−/−^ mice, which have a prominent neuropsychiatric phenotype, providing additional evidence of myelination alterations in ganglioside deficiency [56].

## 6. Gangliosides in Insulin Signalling

Gangliosides are also known to regulate IR function and dysregulation of IR was shown to be associated with increased incidence of ADHD and ASD [100,101]. Ganglioside GM3 was shown to regulate IR activation in membrane microdomains by suppressing its phosphorylation [9] and may mediate the inhibitory effects of TNF on the IR [81].

In our recent study, we found decreased expression of IRs in the spleen and the liver of *St3gal5*^−/−^ male mice accompanied by an increase in body weight, suggesting metabolic changes [57]. Previously, changes in IR signalling in adipose tissue and skeletal muscle were also found in these mice [51]. Given that insulin signalling changes are implicated in neuropsychiatric disorders in multiple pathways, including neuroinflammation and oxidative stress [100,102,103], such changes may also contribute to pathology caused by ganglioside deficiency. It was also shown that insulin signalling may regulate the myelination process. Mice with insulin-resistant Schwann cells due to Schwann cell-specific knockout of IR and insulin-like growth factor receptor 1 (*IGFR1*) showed thinner myelination and the authors hypothesized that insulin resistance in myelinating cells is one of the pathological contributors to diabetic neuropathy [104].

**Figure 2:**
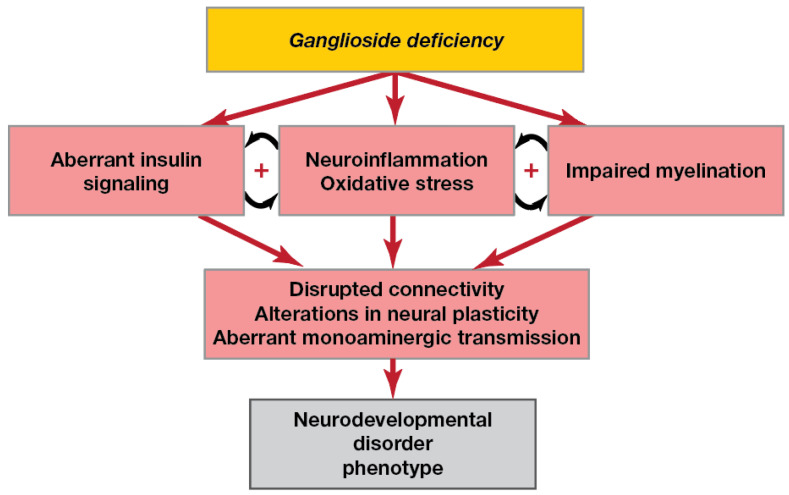
Pathological pathways in brain ganglioside deficiency. Ganglioside deficiency may lead to both neuroinflammation, aberrant insulin signalling and myelination deficits, which are known to be interrelated. Impaired myelination causes neuroinflammation: myelin debris, products of neuron elimination due to necrosis and apoptosis and dysregulation of activity-dependent astrocytes may activate microglia and macrophages. Pro-inflammatory cytokines and dysregulation of glia by inflammation negatively affect myelination. Aberrant signal propagation, axon degradation and neuronal death due to a lack of metabolic support from myelin sheaths disrupt brain connectivity. Additionally, neuroinflammation contributes to alterations in monoaminergic transmission and neural plasticity.

Thus, the available literature suggests that in ganglioside deficiency, interrelated mechanisms such as neuroinflammation, aberrant insulin signalling and impaired myelination occur (Figure 2).

Oxidative stress, caused by reactive oxygen species, is tightly interconnected with neuroinflammation. Oxidative stress in connection with neuroinflammation was also implicated in animal models of various pathologies, such as autism [105], fragile X syndrome [106], Alzheimer’s disease [77,107], amyotrophic lateral sclerosis [79,108] and myelination abnormalities in schizophrenia [109] and other conditions [110].

In patients with autism, antioxidant enzymes have decreased activity in the brain, which is associated with increased levels of lipid peroxidation products [111,112]. In turn, several studies have shown that gangliosides prevent lipid peroxidation product accumulation in synaptosomes and protect synaptosomal cAMP phosphodiesterase and Na^+^, K^+^-ATPase from deactivation caused by lipid peroxidation [111,113]. Increased catalase (CAT) activity was also shown in GM1-treated rat cortex ex vivo, which also may lower oxidative stress [114]. Also, levels of CAT, superoxide dismutase (SOD) and glutathione peroxidase (GSH POD) were increased in primary and peri-ischemic rat cortex treated with GM1 [115]. Exogenously added gangliosides were shown to reduce ROS levels, which are associated with oxidative stress in isolated cell cultures of the heart [116], hepatocytes [117], spermatozoa [118] and PC12 cell culture [119,120]. The aforementioned studies included gangliosides downstream of ST3GAL5 and B4GALNT1. Together, these data suggest that in ganglioside deficits, including those caused by the dysfunction of ST3GAL5 and/or B4GALNT1, the ability to fight inflammation and oxidative stress is compromised.

## 7. Perspectives of Antioxidative Stress and Anti-Inflammatory Manipulation in the Treatment of Ganglioside Deficit-Related Disorders

Since gangliosides have antioxidant and anti-inflammatory effects; the medication that increases the level of ganglioside may be efficient in conditions to which pathogenesis neuroinflammation contributes. One example is resveratrol, a phytoestrogen with antioxidant, anti-inflammatory and neuroprotective effects, which are properties that were studied in the rat model of global cerebral ischemia that leads to an increase in lipid peroxidation and ROS formation. In a model of global cerebral ischemia in rats, a significant decrease in ganglioside, phospholipids and cholesterol in the hippocampus and cortex was observed. Resveratrol administration seven days prior to ischemia prevented the reduction in the total ganglioside content in the hippocampus and cerebral cortex, thus stabilizing disrupted antioxidant defences [121]. Another example of a medication acting on gangliosides is ferulic acid, which was shown to exert an antioxidative effect in Fe^2+^-induced oxidative injury in rat brains. The induction of oxidative injury with FeSO_4_ led to the complete depletion of ganglioside GT3 and ganglioside GD2 and treatment with ferulic acid led to the restoration of GT3 levels [122].

An alternative drug strategy for neuroinflammation regulation could be using gangliosides themselves as a therapy. It was shown in a model of *B4galnt1*^+/−^ mice, which have a partial deficit of major brain gangliosides and demonstrate some of the motor and cognitive features of Parkinson’s disease. Daily intraperitoneal administration of *E. coli* derived GM1 ganglioside alleviated both motor and cognitive deficits in these mice [50]. In addition, authors also demonstrated GM1 uptake by the brain of *B4galnt1*^−/−^ mice upon intraperitoneal administration [50], which suggests that the compound may penetrate the blood–brain barrier and act in the CNS directly with no additional delivery system. Moreover, it was shown that administration of GM1 decreases oxidative stress. In rats in a hypobaric hypoxic (HH) environment, GM1 ganglioside treatment led to the cessation of increased ROS production, which was otherwise observed in this environment without treatment. GM1 also suppressed the accumulation of a marker of oxidative stress, malondialdehyde, and increased levels of SOD and glutathione. Simultaneously, GM1 administration counteracted enhanced inflammation in HH-exposed rats by muting pro-inflammatory cytokines IL-1β, TNF-α and IL-6 levels in serum and brain tissues [123]. Another study in a focal cortical ischemia rat model investigated the changes in the levels of the antioxidant enzymes SOD, GSH POD and CAT. Intramuscular GM1 injection within 10 min of the ischemia increased levels of SOD, CAT and GSH POD, which remained significantly high even at 2 weeks after ischemia [115].

These findings raise the question of whether anti-inflammatory and antioxidant therapies, as well as treatment with gangliosides per se, may be beneficial in disorders associated with or caused by ganglioside deficiency. Clinical data suggests such an approach is viable. Gangliosides combined with mouse nerve growth factor (NGF) showed high therapeutic efficacy in infants with hypoxic-ischemic encephalopathy (HIE). Severe hypoxia in infants with HIE can cause vascular endothelial cells and brain neuron damage by exposure to free radical oxidative stress and release of inflammatory factors, cytokines and immune molecules such as sICAM-1, TNF and interleukins. This study demonstrated the decline of pro-inflammatory factor levels and free radical levels in the treated group, indicating that ganglioside combined with NGF could enhance immune function, improve vascular injury and promote inflammation regression [124]. In a recent meta-analysis of antioxidative interventions in autism, the authors analysed 12 publications where effects of antioxidants such as n-Acetyl-cystein, melatonin, folic acid, arachidonic acid, docosahexaenoic acid, resveratrol, palmitoyl-ethanolamide and sulforaphane were analysed. They concluded that, while additional research is needed, their analysis suggested a potential role of antioxidants in the management of some symptoms of ASD [125]. This suggests potential benefits of a broad range of treatments with anti-oxidant activity, e.g., thiamine compounds and phyto anti-oxidants [126,127,128,129,130]. However, none of the analysed papers included an analysis of ganglioside deficiency. Based on the present critical literature review that evidences a connection between gangliosides, oxidative stress and psychiatric disorders, we may speculate that antioxidant and anti-inflammatory therapies, as well as treatment with gangliosides, should be studied in pathologies associated with ganglioside deficiency.

## 8. Conclusions

Together, clinical and preclinical data suggest that ganglioside metabolism alterations may be involved in a broader spectrum of disorders than was thought previously. A “triad” of inter-related molecular mechanisms of neuroinflammation, myelination and IR signalling might underlie the consequences of compromised ganglioside metabolism contributing to the pathophysiology of neuropsychiatric disorders and might be considered a pattern response to a ganglioside-associated deficit. These findings warrant further studies exploring the role of gangliosides in the diagnosis and pathophysiology and treatment of neuropsychiatric and CNS disorders, including those for which the role of ganglioside dysfunction is not known.
